# Supplementary material for: Rapid turnover of life-cycle-related genes in the brown algae
Source: Genome Biol. 2019 Feb 14;20:35. doi: 10.1186/s13059-019-1630-6 (PMC6374913; doi:10.1186/s13059-019-1630-6)
Supplement: Supplementary file 3 — Protein alignments, corresponding Gblocks html file and CDS sequences for the lineage conserved gametophyte-biased genes. (ZIP 2109 kb) [file 13059_2019_1630_MOESM3_ESM.zip › Con_GA_alignments/Ec-12_004500.aa_ali.fasta-gb.htm]

Ec-12\_004500.aa.fasta.trans.aa\_ali.fasta


## Gblocks 0.91b Results

Processed file: **Ec-12\_004500.aa.fasta.trans.aa\_ali.fasta**  
Number of sequences: **2**  
Alignment assumed to be: **Protein**  
New number of positions: **278** (selected positions are underlined in blue)

```
                         10        20        30        40        50        60
                 =========+=========+=========+=========+=========+=========+
Ec-12_004500     ------------------------------------MLCPAYERGK-------------T
MSTRG.36110.1    XVKQFDVSRRSREMDPVLMRHRNPDVEANRLVREEERVRAAINNAKSRQLRVEQDYDIVT
                                                                            #


                         70        80        90       100       110       120
                 =========+=========+=========+=========+=========+=========+
Ec-12_004500     HLPWNPADVPPPTYVKQFDVSRKS--------RE-------------------MDPVLM-
MSTRG.36110.1    HSSFLPEKQHPPTRPVRHPDTRTSYDILNHVPRDKAHSVALIYGEGEGPEVLPQKAVLVE
                 ########################                                    


                        130       140       150       160       170       180
                 =========+=========+=========+=========+=========+=========+
Ec-12_004500     ----------------RHRFLEKHEERMTEEKRFLRERAEEKYRRTHDYNIIAGAYYDQD
MSTRG.36110.1    GGKKPHSHLKRDVDVLSNRFLEKHEERMVEEQRFLRESAEEKYRRTHDYNIIAGTFYDRN
                                   ##########################################


                        190       200       210       220       230       240
                 =========+=========+=========+=========+=========+=========+
Ec-12_004500     KERKFVNSRDKLLTMQGRAQQYRLPPSIRYGEGNGYNIINQQPKKTVVDMTSASVQDRGL
MSTRG.36110.1    KERDFVDSREKLEAMQGRAQQYRFPPSMRYGEGNDYNIINQEPKKTVVKNKPASVADRGL
                 ############################################################


                        250       260       270       280       290       300
                 =========+=========+=========+=========+=========+=========+
Ec-12_004500     SKRTRGRQTYEDNIRQHERAAAASEQRTLNRPSHMRHVEVLRTGHNVISNQDFAGREGKP
MSTRG.36110.1    SKRTRGRQAHEENIRRHERAAALGEERTLNRPSHMRHVEVLRTGHNVVSNEVFTGREGKP
                 ############################################################


                        310       320       330       340       350       360
                 =========+=========+=========+=========+=========+=========+
Ec-12_004500     PPQPRAKPPMPVWDRLKGKHEMPEAAAIGSMPTAETNDGGQQHGDVTGLGQTNSRDAGGS
MSTRG.36110.1    PPQPRARPPVPVWDRLSGQSKTSRSTADTWKKQPE-----EEHRACGGNDRASSR-----
                 ###################################       #############     


                        370       380       390       400       410       420
                 =========+=========+=========+=========+=========+=========+
Ec-12_004500     SGRCGGQVPASPGGARGTEQPGTRREAPPPLREGSSNTGGRIDDHGGETNRGIGGRP-SA
MSTRG.36110.1    ----GGQ----------------PRQAPGGDGEGDVH--PGVSDGDREERGVWAGRPDAA
                                         ##########         ##############  #


                        430       440
                 =========+=========+==
Ec-12_004500     EAAMPSTKPAVPPLNLSRS---
MSTRG.36110.1    TGEIPGSRPPVPPLDLSRRPET
                 ##################
```

```
Parameters used
Minimum Number Of Sequences For A Conserved Position: 2
Minimum Number Of Sequences For A Flanking Position: 2
Maximum Number Of Contiguous Nonconserved Positions: 8
Minimum Length Of A Block: 10
Allowed Gap Positions: None
Use Similarity Matrices: Yes
```

```
Flank positions of the 6 selected block(s)
Flanks: [60  84]  [139  335]  [343  355]  [385  394]  [404  417]  [420  438]  

New number of positions in Ec-12_004500.aa.fasta.trans.aa_ali.fasta-gb:  278  (62% of the original 442 positions)
```
